# Response of Different Insect Groups to Various Wavelengths of Light under Field Conditions

**DOI:** 10.3390/insects12050427

**Published:** 2021-05-10

**Authors:** Hongsheng Pan, Gemei Liang, Yanhui Lu

**Affiliations:** 1 State Key Laboratory for Biology of Plant Diseases and Insect Pests, Institute of Plant Protection, Chinese Academy of Agricultural Sciences, Beijing 100193, China; panhongsheng0715@163.com (H.P.); gmliang@ippcaas.cn (G.L.); 2 Scientific Observing and Experimental Station of Crop Pests in Korla, Ministry of Agriculture and Rural Affairs, Institute of Plant Protection, Xinjiang Academy of Agricultural Sciences, Urumqi 830091, China

**Keywords:** pest, natural enemy, insect family, light trap, population monitoring, pest management

## Abstract

**Simple Summary:**

Laboratory experiments have found that insects in the same taxonomic group generally have similar responses to light at various wavelengths. However, there is lack of direct evidence of between-group differences in insect responses to various light wavelengths under field conditions. During 2014–2015, the relative attractiveness of LEDs with 19 single wavelengths to three pest orders and four natural predator orders was evaluated in cotton fields. The average numbers of Lepidoptera, Hemiptera, Coleoptera, and total pests captured by traps with a 395-nm LED wavelength were higher than those for all others, except 440-nm wavelength captured the largest number of Hemiptera in 2015. For natural enemies, the average numbers of Coleoptera, Neuroptera, and total natural enemies were the largest in traps with a 572-nm LED wavelength, except 538-nm wavelength captured the largest number of Coleoptera in 2014. In general, the ratio of pests to natural enemies captured in the 395-nm wavelength LED trap was significantly more than all others. These results demonstrated that insects in different taxonomic groups have significantly different responses to light at various wavelengths under field conditions; these results will provide insights for in-depth studies on insect phototaxis and guide the long-term monitoring of insects in different groups.

**Abstract:**

Insects in the same taxonomic group generally have similar responses to light at various wavelengths in the laboratory. However, there is lack of direct evidence of between-group differences in insect responses to various light wavelengths under field conditions. During 2014 and 2015, we evaluated the relative attractiveness of LEDs with 19 single wavelengths to three pest orders and four natural predator orders in cotton fields. The average numbers of Lepidoptera, Hemiptera, Coleoptera, and total pests captured by traps with a 395-nm LED wavelength were higher than those for all others, except 440-nm wavelength captured the largest number of Hemiptera in 2015. For natural enemies, the average numbers of Coleoptera, Neuroptera, and total natural enemies were the largest in traps with a 572-nm LED wavelength, except 538-nm wavelength captured the largest number of Coleoptera in 2014. In general, the ratio of pests to natural enemies captured in the 395-nm wavelength LED trap was significantly more than all others. These results demonstrated that insects in different taxonomic groups have significantly different responses to light at various wavelengths under field conditions; these results will provide insights for in-depth studies on insect phototaxis and guide the long-term monitoring of insects in different groups.

## 1. Introduction

The insect visual system can sense external light stimulation, which plays an important role in insect foraging, communication, courtship, and avoidance of natural enemies [1]. Over the long course of evolution, insects have developed phototropic behaviors in response to light sources, including positive phototropic behavior toward light sources and negative phototropic behavior away from light sources [2]. Light trapping technology based on insect phototactic behavior has been widely used in the field monitoring of agricultural pests (e.g., noctuid moths, weevils, and scarab beetles), the establishment of early warning systems, and even the development of ecofriendly prevention and control strategies [3,4,5,6,7,8].

The sensitive visual spectrum in insects is mostly concentrated in the wavelength range of 253–700 nm, and a large number of laboratory experiments have found that insects in the same taxonomic group generally have similar responses to light of different wavelengths and preferences for wavelength ranges [2,9]. For example, electrophysiological recordings in 35 species of Lepidoptera showed that they responded to wavelengths of 360–400, 420–460, and 520–560 nm [10], and the larvae of lepidoptera investigated thus far showed responses in three spectrum ranges: 340–370 nm, 440–450 nm, and 520–540 nm [11,12,13]. However, there is much variation between different taxonomic groups [14]; polyphagous aphids, for example, were significantly attracted to lights with wavelengths ranging from 490 to 550 nm [15]. In addition, several field trials showed that shorter-wavelength lights attracted significantly more Noctuidae than longer-wavelength lights, while both wavelengths were equally attractive to Geometridae [16]. The trapping effects of four wavelengths adjacent to a black light lamp (365 ± 50 nm), i.e., 375 nm, 385 nm, 395 nm, and 405 nm, on five adult pests (i.e., *Helicoverpa armigera*, *Mythimna separata*, *Athetis lepigone*, *Anomala corpulenta,* and *Holotrichia parallela*) were compared in cotton fields during 2017, and the 385-nm LED lamp had the best trapping effect [17]. However, in general, there is still a lack of direct evidence of differences between different groups of insects exposed to light with various wavelengths under field conditions.

In China, light trapping of important crop insect pests, such as *H. armigera* and *M. separata*, has been annually conducted nationwide for >30 years (since the late 1980s) and has provided important data for pest forecasting. Several light devices, such as incandescent lamps, black light lamps, and two-wave lamps, have been widely used in China [7]. The light-traps mentioned above have different materials and light sources; however, they have relatively concentrated and similar wavelength ranges [18,19,20,21]. Recently, developed LED technology has advanced insect traps and improved trap efficiency by using less energy and producing narrow-spectral bands of light [22,23,24,25,26]. If different insect groups show various preferences for light wavelengths under field conditions, such a replacement of traditional light-traps by LED traps will have a significant impact on long-term and area-wide monitoring of insects (e.g., crop pests) with light-traps and the further use of historical datasets in pest population forecasting.

To test the hypothesis that different insect groups respond differently to different wavelengths, we assessed the relative attractiveness of LEDs with various wavelengths to pests and their natural enemies in different taxonomic groups in cotton fields during 2014–2015. Our work will advance the in-depth understanding of insect phototaxis and provide a scientific basis for the development of light-trap technology and comparative analyses of different light-trap results.

## 2. Materials and Methods

### 2.1. Field Trials

During 2014 and 2015, field trials were conducted from July to September when higher population abundances of pests and their natural enemies were observed in 5-ha cotton fields at the Xinxiang Experiment Station, Chinese Academy of Agricultural Sciences (CAAS; 35.15° N, 113.80° E) (Xinxiang, China). Specifically, the relative attractiveness of cotton pests and their natural enemies was assessed using traps equipped with 5-W LEDs at 19 single wavelengths from the insect-visible range: 375, 395, 418, 440, 460, 484, 506, 528, 538, 550, 572, 594, 616, 638, 660, 682, 704, 726, and 748 nm. The LED traps were purchased from Xinxiang Tianyi New Energy Technology Development Co. Ltd. (Xinxiang, China). The LED trap is a cylinder with a diameter of 42.5 cm and a height of 83 cm, is mainly composed of LED lamp, power grid (instantaneous voltage is 2300 V ± 150 V), and plastic bucket for insect collecting. The LED lamp is placed vertically at the top of the lamp, and the outer ring is surrounded by a high-voltage power grid 10 cm away. The plastic bucket for collecting insects is placed below the lamp tube to store the insects caught.

**Figure 1:**
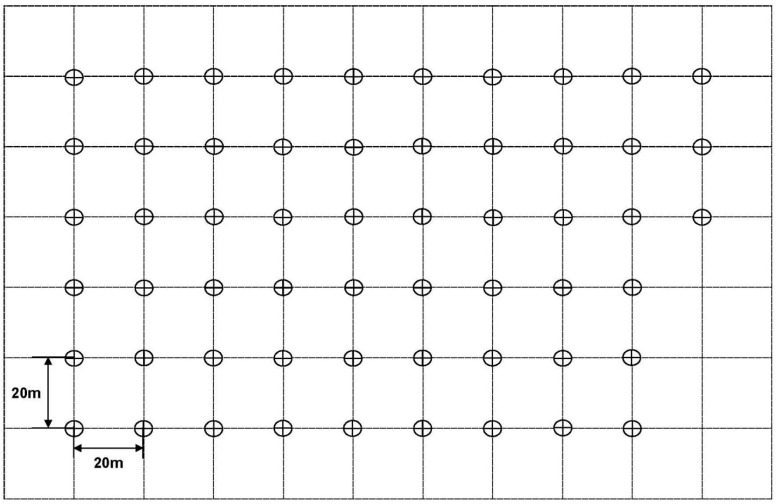
Space layout of 57 LED-equipped traps at 19 single wavelengths in the field. Each circle in the picture represents a trap.

Every year, cotton plants were drill-seeded in mid-April, and no insecticides were used during the field trials. The lights were positioned on top of vertical steel rods at approximately 50 cm above the cotton canopy. The experimental fields were embedded within agriculture-dominated landscapes with comparatively low levels of light pollution. Fields were also located >1 km from the main road to limit interference from street lighting or passing traffic. For each wavelength, a total of three LED traps (i.e., three replicates) were deployed in a randomized complete block design. LED traps with various wavelengths were distributed throughout the cotton field, spaced at approximately 20 m (Figure 1). For safety reasons, LED traps were not used on rainy or windy days. LED traps were operated from 18:00 to 06:00 of the next day. Insects attracted to the LEDs were stunned by a high-voltage grid around the LED and consequently dropped into a mesh bag that was positioned below each LED trap.

Every other day, field-collected samples were processed, and the number of each insect species was determined. Species identification of cotton pests and their natural enemies was performed according to Lu et al. [27] and the Atlas of Cotton Pests and Their Natural Enemies [28]. To be more specific, cotton pests refer to the herbivorous insects that feed on cotton plants, and natural enemies refer to insects that prey on or parasitize the cotton pests.

### 2.2. Statistical Analysis

To meet assumptions of normality and heteroscedasticity, data were square-root transformed (average abundances of pests and natural enemies in different taxonomic groups) or arcsine square-root transformed (the ratio of insect orders or pests to natural enemies). Next, differences between individual LED wavelengths were assessed using one-way analysis of variance (ANOVA), followed by Duncan’s new multiple range test (MRT). For the insect orders of pests and natural enemies, we performed a chi-square test to clarify differences among the 19 tested wavelengths. All statistical analyses were performed using SAS [29].

## 3. Results

### 3.1. Insect Abundances of Pests and Natural Enemies in Different Orders

In the field trial, six main orders, including Lepidoptera, Hemiptera, and Coleoptera for pests and Coleoptera, Neuroptera, Diptera, and Hymenoptera for natural enemies, were recorded (Table 1). Overall, a total of 7467 adults (5828 pests, 1639 natural enemies) and 9511 adults (8866 pests, 645 natural enemies) from the six target orders were trapped in 2014 and 2015, respectively.

**Figure 2:**
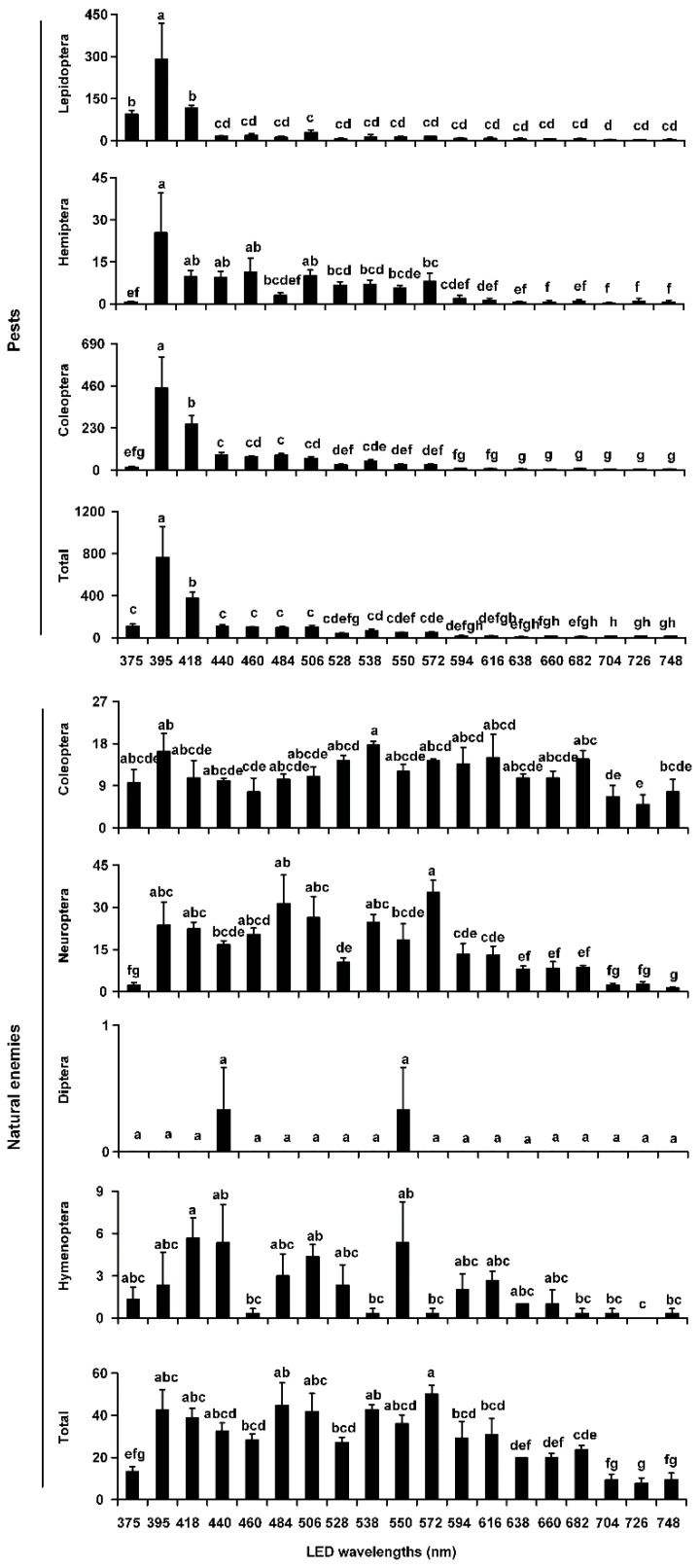
Average abundances of pests and natural enemies in different taxonomic groups trapped by LED-equipped traps with 19 single wavelengths in 2014. The bar charts show the average number of pests or natural enemies caught per trap, as recorded during trials in cotton fields in Xinxiang County (Xinxiang, China). Individual bars represent the mean (±SE) trap capture, with accompanying letters denoting statistically significant differences among the LED wavelengths in a given order or total pests or natural enemies (ANOVA, *p* < 0.05).

In 2014, the average number of Lepidoptera, Hemiptera, Coleoptera, and total pests captured by traps with a 395-nm LED wavelength were higher than those in traps with all other wavelengths (Lepidoptera: *F* = 13.52, *df* = 18, 38, *p* < 0.0001; Hemiptera: *F* = 5.76, *df* = 18, 38, *p* < 0.0001; Coleoptera: *F* = 26.35, *df* = 18, 38, *p* < 0.0001; total pests: *F* = 23.00, *df* = 18, 38, *p* < 0.0001) (Figure 2, Table 2). For natural enemies, the average number of Coleoptera was the largest in traps with a 538-nm LED wavelength, with significant differences among the 19 tested wavelengths (*F* = 1.95, *df* = 18, 38, *p* = 0.0413), and the average numbers of Neuroptera and total natural enemies were the largest in traps with a 572-nm LED wavelength, with significant differences among the 19 wavelengths (Neuroptera: *F* = 9.85, *df* = 18, 38, *p* < 0.0001; total natural enemies: *F* = 7.69, *df* = 18, 38, *p* < 0.0001). There were no significant differences among the 19 tested wavelengths for Diptera and Hymenoptera (all *p* > 0.05) (Figure 2, Table 2).

**Figure 3:**
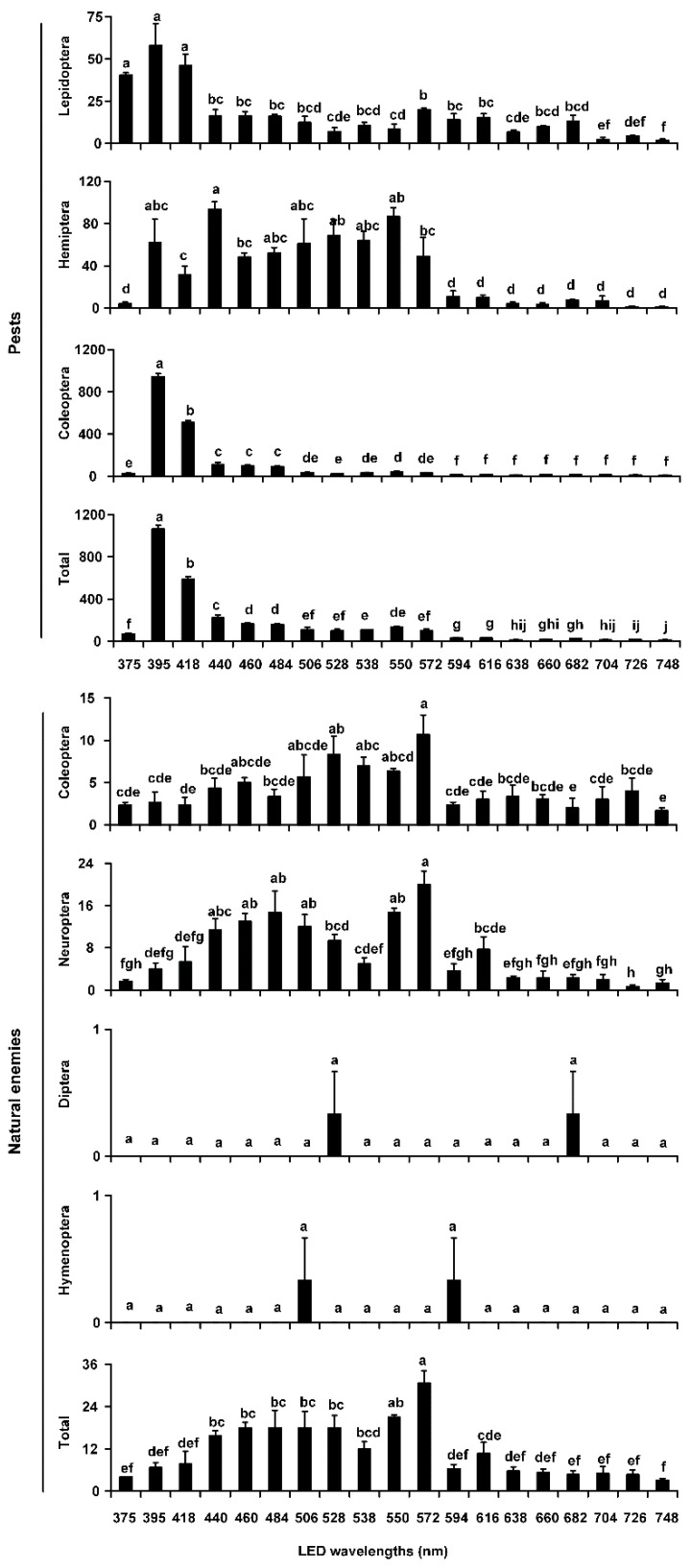
Average abundances of pests and natural enemies in different taxonomic groups trapped by LED-equipped traps with 19 single wavelengths in 2015. Bar charts show the average number of pests or natural enemies caught per trap, as recorded during trials in cotton fields in Xinxiang County (Xinxiang, China). Individual bars represent the mean (±SE) trap capture, with accompanying letters denoting statistically significant differences among the LED wavelengths in a given order or total pests or natural enemies (ANOVA, *p* < 0.05).

In 2015, traps with a 395-nm LED wavelength captured the most lepidopterans, although this number did not significantly differ from traps with 375-nm or 418-nm wavelengths. However, traps with LEDs with the above three wavelengths captured significantly more lepidopterans than traps with all the other wavelengths (*F* = 14.56, *df* = 18, 38, *p* < 0.0001). The average number of Hemiptera captured was the largest in traps with a 440-nm LED wavelength, with significant differences among the 19 wavelengths (*F* = 14.90, *df* = 18, 38, *p* < 0.0001). The average numbers of Coleoptera and total pests captured in LED traps with a 395-nm wavelength were significantly higher than those captured in traps with all other wavelengths (Coleoptera: *F* = 309.25, *df* = 18, 38, *p* < 0.0001; total pests: *F* = 164.39, *df* = 18, 38, *p* < 0.0001) (Figure 3, Table 2). For natural enemies, the average numbers of Coleoptera, Neuroptera, and total natural enemies were the largest in traps with a 572-nm LED wavelength, and there were significant differences among the 19 wavelengths for all (Coleoptera: *F* = 2.71, *df* = 18, 38, *p* = 0.0048; Neuroptera: *F* = 9.57, *df* = 18, 38, *p* < 0.0001; total natural enemies: *F* = 9.38, *df* = 18, 38, *p* < 0.0001). There were no significant differences in the numbers of Diptera and Hymenoptera caught among the 19 wavelengths (all *p* > 0.05) (Figure 3, Table 2).

**Figure 4:**
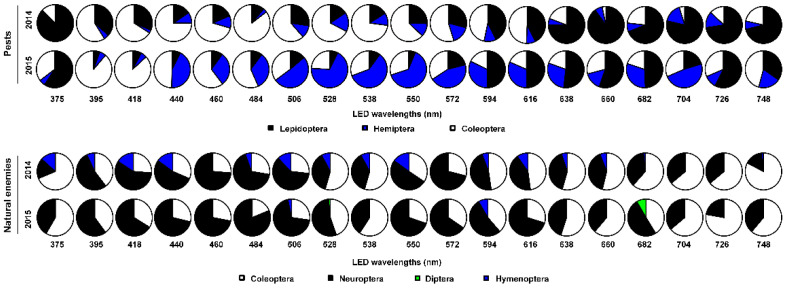
The order compositions of pests and natural enemies trapped in LED-equipped traps with 19 single wavelengths during 2014–2015.

Generally, there were significant differences in the order compositions of captured pests in 2014 (χ^2^ = 29.65, *df* = 2, *p* < 0.0001) and 2015 (χ^2^ = 37.98, *df* = 2, *p* < 0.0001) and in those of natural enemies in 2014 (χ^2^ = 59.79, *df* = 3, *p* < 0.0001) and 2015 (χ^2^ = 71.74, *df* = 3, *p* < 0.0001) (Figure 4).

### 3.2. Ratio of Pests to Natural Enemies

**Figure 5:**
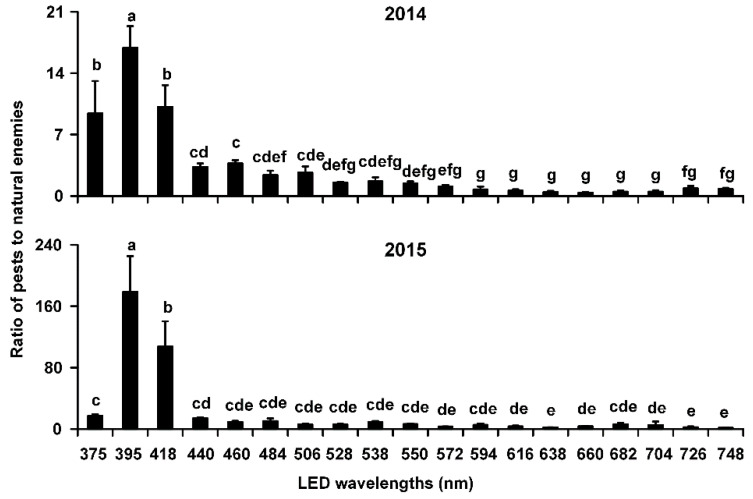
Ratios of pests to natural enemies trapped in LED-equipped traps with 19 single wavelengths during 2014–2015. Individual bars represent the mean (±SE) ratio of pests to natural enemies, with accompanying letters denoting statistically significant differences among the LED wavelengths in a given year (ANOVA, *p* < 0.05).

In 2014 and 2015, the ratios of pests to natural enemies were the highest in LED traps with a 395-nm wavelength (2014: 16.91 ± 2.45; 2015: 178.92 ± 46.37); the ratios were significantly higher than those in traps with all other tested wavelengths (2014: *F* = 21.95, *df* = 18, 38, *p* < 0.0001; 2015: *F* = 22.49, *df* = 18, 38, *p* < 0.0001) (Figure 5).

## 4. Discussion

Usually, insects in the same taxonomic group show similar responses to light with different wavelengths in the laboratory [2,9]. In the present study, the dominant groups with high abundances, namely, Coleoptera (mainly including scarabs) and Lepidoptera (mainly including noctuids), showed obvious and consistent preferences for 395 nm and 418 nm in both years (2014–2015). A total of five and six dominant species of Coleoptera and Lepidoptera were collected, respectively, greatly supporting the above hypothesis. The results were also similar to phototaxis behavioral patterns observed in previous studies on *H. armigera* adults [30] as well as *A. corpulenta* and *H. parallela* adults [31]. However, for the insect groups with low abundance, such as Hemiptera pests and the four orders of natural enemies, peak abundances were not consistent for specific light wavelengths in 2014 and 2015. Hence, further assessments of annual changes in insect abundance and other factors under field conditions are needed.

Although ultraviolet and blue light are usually most attractive to insects, the degree of attraction varies among orders [2,16,32,33,34]. In this study, Coleoptera and Lepidoptera had highly similar wavelength preferences, and LED traps equipped with a 395-nm light were the most attractive to Coleoptera and Lepidoptera; however, traps with a 375-nm light also captured many Lepidopteran adults, though it mostly failed to attract Coleoptera. For the other orders, the differences in insect preferences for specific light wavelengths were greater. This was partly due to between-group differences in phototactic behavior and the relatively low abundance of these insect orders during 2014–2015.

Despite attempts to improve monitoring efficacy for target pest species, insect traps catch many nontarget insects, including beneficial natural enemy species [35]. In this study, we found significant differences in the preferred wavelengths between the pest group and natural enemy group. LED traps with a 395-nm wavelength captured three pest orders throughout the whole growth stage in cotton fields and captured a higher ratio of pests to natural enemies. Thus, this wavelength can increase the efficacy of pest trapping and reduce the negative effect on the population of natural enemies. LED traps will be an important method for monitoring and trapping insect pests, which can minimize the disadvantage of killing natural enemies by traditional light trapping [7,20,21,36]. The optimum wavelength of LED traps mainly depends on the target pest species, natural enemy species, or their orders in specific agroecosystems. For example, Wu et al. [37] reported that timely and proper application of lamps with wavelengths of 390–400, 410–420, 440–445, and 450–460 nm effectively trapped the main insect pests and reduced bycatch of their natural enemies in vegetable fields. Hence, suitable LED light-traps need to be selected and fully assessed in important agroecosystems.

In general, light-traps are specialized devices that are not well suited for quantitative surveys of a wide range of taxa. Light-trap surveys mainly focus on Lepidoptera and Coleoptera, which demonstrate strong phototactic behavior; therefore, light-traps are the most efficient traps for these orders. Ramamurthy et al. [14] studied the different numbers of insect species caught by light-traps with different light sources and reported that Coleopterans dominated the catches, consistent with our present study in which Coleoptera accounted for more than 60% of the trapped pests during 2014–2015. Hence, the phototactic behavior of Lepidoptera and Coleoptera in response to different LED wavelengths or various combinations of LED wavelengths in the field should be further assessed. This is an important topic for the development of LED trapping techniques for insect light trapping and monitoring.

When choosing an LED light source to trap pests, the first thing to do is to choose the appropriate wavelength regions [2,7,21]. The biggest problem of traditional light-traps is that it can kill not only insect pests but also natural enemies [20,36]. Ladybeetles preferred the blacklight lamp, and meanwhile, *Ophion* sp. (Hymenoptera: Ichneumonidae) was significantly more abundant in that lamp [38]. *Harmonia axyridis* showed notably preference to color with green-yellow wavelengths between 500–600 nm [39], which was consistent with the present study; that is, 538 nm and 572 nm all belonged to that region. In this study, LED traps with a 395-nm wavelength captured a higher ratio of pests to natural enemies. Thus, LED light with 395-nm has obvious selectivity to natural enemies (e.g., ladybeetles, lacewings, and parasitoids).

In northern China, insects of at least 75 families from 13 orders had been trapped by conventional black light lamps [40]. We also trapped great numbers of species of insects in the LED traps in cotton fields in this study, but only studied some dominant pest and natural enemy species with larger body sizes, and relatively little attention has been given to vast species of secondary and smaller-bodied insects. This is a topic that needs further strengthening and exploration.

## 5. Conclusions

Our results suggest that there were significant differences between insect groups in their responses to various light wavelengths, and the insect compositions greatly differed among the different LED traps with single wavelengths under field conditions. Before the wide-scale replacement of traditional traps with wide wavelengths can be implemented, more comparative analyses comparing traditional traps with LED traps with specific wavelengths need to be conducted. This data will provide a necessary basis for long-term monitoring data and precise insect forecasting, which is an important part of integrative pest management in China.

## Figures and Tables

**Table 1:** Insect species surveyed in the LED traps with 19 single wavelengths during 2014–2015.

Category	Orders	Insect Species
Pests	Lepidoptera	*Helicoverpa armigera* Hübner
		*Agrotis ypsilon* Rottemberg
		*Spodotera exigua* Hübner
		*Scotogramma trifolii* (Rottemberg)
		*Macdunnoughia crassisigna* (Warren)
		*Argyrogramma agnata* (Staudinger)
	Hemiptera	*Apolygus lucorum* (Meyer-Dür)
		*Adelphocoris suturalis* Jakovlev
		*Adelphocoris fasciaticollis* Reuter
		*Dolycoris baccarum* Linnaeus
		*Empoasca biguttula* Ishida
	Coleoptera	*Holotrichia parallela* Motschulsky
		*Holotrichia oblita* Faldermann
		*Holitrichia trichophora* (Fairmaire)
		*Anomala corpulenta* Motschulsky
		*Monolepta hieroglyphica* Motschulsky
Natural enemies	Coleoptera	*Propylea japonica* Thunberg
		*Harmonia axyridis* Pallas
	Neuroptera	Lacewings
	Diptera	Hoverflies
	Hymenoptera	Parasitoid wasps

**Table 2:** Statistical values from the average abundance analyses of pests and natural enemies in different taxonomic groups trapped in LED traps with 19 single wavelengths during 2014–2015.

Year	Category	Groups	Statistical Values
2014	Pests	Lepidoptera	*F* = 13.52, *df* = 18,38, *p* < 0.0001
		Hemiptera	*F* = 5.76, *df* = 18,38, *p* < 0.0001
		Coleoptera	*F* = 26.35, *df* = 18,38, *p* < 0.0001
		Total	*F* = 23.00, *df* = 18,38, *p* < 0.0001
	Natural enemies	Coleoptera	*F* = 1.95, *df* = 18,38, *p* = 0.0413
		Neuroptera	*F* = 9.85, *df* = 18,38, *p* < 0.0001
		Diptera	*F* = 0.94, *df* = 18,38, *p* = 0.5363
		Hymenoptera	*F* = 1.77, *df* = 18,38, *p* = 0.0680
		Total	*F* = 7.69, *df* = 18,38, *p* < 0.0001
2015	Pests	Lepidoptera	*F* = 14.56, *df* = 18,38, *p* < 0.0001
		Hemiptera	*F* = 14.90, *df* = 18,38, *p* < 0.0001
		Coleoptera	*F* = 309.25, *df* = 18,38, *p* < 0.0001
		Total	*F* = 164.39, *df* = 18,38, *p* < 0.0001
	Natural enemies	Coleoptera	*F* = 2.71, *df* = 18,38, *p* = 0.0048
		Neuroptera	*F* = 9.57, *df* = 18,38, *p* < 0.0001
		Diptera	*F* = 0.94, *df* = 18,38, *p* = 0.5363
		Hymenoptera	*F* = 0.94, *df* = 18,38, *p* = 0.5363
		Total	*F* = 9.38, *df* = 18,38, *p* < 0.0001

## Data Availability

All data analyzed in this study are included in this article.

## References

[1] Chapman R.R. (1998). The Insects: Structure and Function.

[2] Briscoe A.D., Chittka L. (2001). The evolution of color vision in insects. Annu. Rev. Entomol..

[3] Song X.Y., Zhang G.X., Li X.J. (2005). Application of frequency-vibrancy pest-killing lamp in controlling agricultural pests. Crops.

[4] Cowan T., Gries G. (2009). Ultraviolet and violet light: Attractive orientation cues for the Indian meal moth, *Plodia interpunctella*. Entomol. Exp. Appl..

[5] Fan X.Y., Li Y.Z., Wang X.L., Lin L.J., Yu H.L., Guan J.C. (2009). Experiment on attraction of scarab adults (Scarabaeidae) to a black light lamp in the Chinese olive (*Canarium album*) plantation. Guangdong Forest Sci. Tech..

[6] Shimoda M., Honda K.-I. (2013). Insect reactions to light and its applications to pest management. Appl. Entomol. Zoöl..

[7] Sang W., Huang Q.Y., Wang X.P., Guo S.H., Lei C.L. (2019). Progress in research on insect phototaxis and future prospects for pest light-trap technology in China. Chin. J. Appl. Entomol..

[8] Beck J., Linsemair K.E. (2006). Feasibility of light-trapping in community research on moths: Attraction radius of light, completeness of samples, nightly flight times and seasonality of Southeast-Asian hawkmoths (Lepidoptera: Sphingidae). J. Res. Lepid..

[9] Prokopy R.J., Owens E.D. (1983). Visual Detection of Plants by Herbivorous Insects. Annu. Rev. Entomol..

[10] Eguchi E., Watanabe K., Hariyama T., Yamamoto K. (1982). A comparison of electrophysiologically determined spectral responses in 35 species of Lepidoptera. J. Insect Physiol..

[11] Gilbert C. (1994). Form and function of stemmata in larvae of holometabolous insects. Annu. Rev. Entomol..

[12] Lin J.T., Hwang P.C., Tung L.C. (2002). Visual organization and spectral sensitivity of larval eyes in the moth *Trabala vishnou*, Lefebur (Lepidoptera: Lasiocampidae). Zool. Stud..

[13] Castrejon F., Rojas J.C. (2010). Behavioral responses of larvae and adults of *Estigmene acrea* (Lepidoptera: Arctiidae) to light of dif-ferent wavelengths. Fla. Entomol..

[14] Ramamurthy V.V., Akhtar M.S., Patankar N.V., Menon P., Kumar R., Singh S.K., Ayri S., Parveen S., Mittal V. (2010). Efficiency of different light source in light traps in monitoring insect diversity. Munis Entomol. Zool..

[15] Fu G.X., Li W.Z., Wu S.Y., Yuan G.H., Wang Y.H., An J.J., Chai X.L. (2009). Bioassays on phototactic responses of *Myzus persicae* (Homoptera: Aphididae) to different monochromatic lights. Acta Entomol. Sin..

[16] Somers-Yeates R., Hodgson D., McGregor P.K., Spalding A., Ffrench-Constant R.H. (2013). Shedding light on moths: Shorter wavelengths attract noctuids more than geometrids. Biol. Lett..

[17] Xu Y.L. (2020). Field Evaluation of Light-Emitting Diodes with Different Wave Lengths as Traps of Five Agricultural Pests.

[18] Zhang G.X., Zheng G., Li X.J., Bu J. (2004). Discussion of using frequency trembler grid lamps from angle of protecting biodiversity. Entomol. Knowl..

[19] Duehl A.J., Cohnstaedt L.W., Arbogast R.T., Teal P. (2011). Evaluating light attraction to increase trap efficiency for *Tribolium castaneum* (Coleoptera: Tenebrionidae). J. Econ. Entomol..

[20] Bian L., Sun X.L., Gao Y., Luo Z.X., Jin S., Zhang Z.Q., Chen Z.M. (2012). Research on the light tropism of insects and the progress in application. Chin. J. Appl. Entomol..

[21] Yang X.M., Lu Y.H., Liang G.M. (2020). Insect phototaxis behavior and light trapping technology. Zhaoming Gongcheng Xuebao.

[22] Nakamoto Y., Kuba H. (2004). The effectiveness of a green light emitting diode (LED) trap at capturing the West Indian sweet potato weevil, *Euscepes postfasciatus* (Fairmaire) (Coleoptera: Curculionidae) in a sweet potato field. Appl. Èntomol. Zoöl..

[23] Kim M.-G., Lee H.-S. (2013). Attractive Effects of American Serpentine Leafminer, *Liriomyza trifolii* (Burgess), to Light-Emitting Diodes. J. Insect Behav..

[24] Park J.-H., Sung B.-K., Lee H.-S. (2015). Phototactic behavior 7: Phototactic response of the maize weevil, *Sitotroga zeamais* motsch (Coleopter: Curculionidae), to light-emitting diodes. J. Korean Soc. Appl. Biol. Chem..

[25] Xu X., Ma L., Yue Z.L., Chen H.B. (2017). Trapping effect of LED single wavelength insecticidal lamp against tea insect pests. China Plant Prot..

[26] Sang W., Cai F.Y., Wang X.P., Zhang S., Huang Q.Y., Zhu F., Guo S.H., Lei C.L. (2018). Application status and prospects of insect trapping lamp in fields. China Plant Prot..

[27] Lu Y.H., Jian G.L., Wu K.M. (2013). Concise Identification Manual of Main Diseases and Insect Pests of Cotton.

[28] Institute of Plant Protection, Hubei Academy of Agricultural Sciences (1980). Atlas of Cotton Pests and Their Natural Enemies.

[29] SAS Institute (2005). SAS/STAT User’s Guide, version 9.13.

[30] Pan H., Xu Y., Liang G., Wyckhuys K.A., Yang Y., Lu Y. (2020). Field evaluation of light-emitting diodes to trap the cotton bollworm, *Helicoverpa armigera*. Crop. Prot..

[31] Xu Y.L., Pan H.S., Liang G.M., Yang Y.Z., Lu Y.H. (2020). Field evaluation of light-emitting diodes with different wavelengths as traps of *Anomala corpulenta* and *Holotrichia parallela*. Xinjiang Agri. Sci..

[32] Poiani S., Dietrich C., Barroso A., Costa-Leonardo A. (2014). Effects of residential energy-saving lamps on the attraction of nocturnal insects. Light. Res. Technol..

[33] Van Grunsven R.H.A., Donners M., Boekee K., Tichelaar I., Van Geffen K.G., Groenendijk D., Berendse F., Veenendaal E.M. (2014). Spectral composition of light sources and insect phototaxis, with an evaluation of existing spectral response models. J. Insect Conserv..

[34] Longcore T., Aldern H.L., Eggers J.F., Flores S., Franco L., Hirshfield-Yamanishi E., Petrinec L.N., Yan W.A., Barroso A.M. (2015). Tuning the white light spectrum of light emitting diode lamps to reduce attraction of nocturnal arthropods. Philos. Trans. R. Soc. B Biol. Sci..

[35] Spears L.R., Ramirez R.A. (2015). Learning to love leftovers: Using bycatch to expand our knowledge in entomology. Am. Entomol..

[36] Zhao J.W., He Y.X., Weng Q.Y. (2008). Application and research of insect light traps in China. Entomol. J. East China.

[37] Wu S., Zhang Y.M., Guo X., Huang Y.F., Liu J.F. (2021). Effectiveness of different wavelength LED insect lamp traps in vegetable fields. Chin. J. Appl. Entomol..

[38] Nabli H., Bailey W.C., Necibi S. (1999). Beneficial Insect Attraction to Light Traps with Different Wavelengths. Biol. Control..

[39] Mondor E.B., Warren J.L. (2000). Unconditioned and conditioned responses to colour in the predatory coccinellid, *Harmonia axyridis* (Coleoptera: Coccinellidae). Eur. J. Entomol..

[40] Cheng D.F., Feng H.Q., Wu K.M. (2005). Scanning Insect Radar and Insect Migration Monitoring.

